# Current News

**Published:** 1898-03

**Authors:** 


### Current News.

### THE NEW YORK INSTITUTE OF STOMATOLOGY.

The following officers were elected for 1898: President, Dr. E. A. Bogue; Vice-President, Dr. C. A. Woodward; Recording Secretary, Dr. F. Milton Smith; Corresponding Secretary, Dr. George A. Wilson; Treasurer, Dr. J. A. Bishop; Editor, Dr. S. E. Davenport; Curator, Dr. J. G. Palmer.

*Executive Committee.*—Dr. A. H. Brockway, Dr. C. O. Kimball, Dr. J. Morgan Howe, Chairman.

### NINTH INTERNATIONAL CONGRESS OF HYGIENE AND DEMOGRAPHY.

There will be held in Madrid, Spain, April, 1898, an International Congress, devoted to the "discussion of questions appertaining to individual and collective hygiene, and to the science of population. It will consist of lectures, the reading of themes, discussions, and practical demonstrations with new instruments and apparatus. The governments of all countries, the provincial and municipal administrative corporations, the universities, academies, schools, and scientific societies that occupy themselves in questions bearing relation to hygiene and demography are invited to give their help to the congress and to send delegates to be their representatives in it."

Those desiring further information can apply to the Secretary-General, Dr. Amalio Gimeno, Madrid, Spain.

### RHODE ISLAND DENTAL SOCIETY.

At a meeting of this Society, the resolutions of the American Academy of Dental Science, Boston, Mass. (International Dental Journal, May 1897), relating to the character of the advertisements appearing in some of the self-styled dental journals were re-adopted by this body, and the secretary was directed to forward this decision to the dental journals.

Clarence A. Carr,
*Secretary.*

